# Postpartum Weight Retention and Cardiometabolic Risk among Saudi Women: A Follow-Up Study of RAHMA Subcohort

**DOI:** 10.1155/2019/2957429

**Published:** 2019-07-01

**Authors:** Hayfaa A. Wahabi, Amel A. Fayed, Shabana Tharkar, Samia A. Esmaeil, Hanadi Bakhsh

**Affiliations:** ^1^Chair of Evidence-Based Healthcare and Knowledge Translation, College of Medicine, King Saud University, Riyadh, Saudi Arabia; ^2^Department of Family and Community Medicine, College of Medicine, King Saud University, Riyadh, Saudi Arabia; ^3^College of Medicine, Princess Nourah Bint Abdulrahman University, Riyadh, Saudi Arabia; ^4^Department of Biostatistics, High Institute of Public Health, Alexandria University, Alexandria, Egypt; ^5^Prince Sattam Chair for Epidemiology and Public Health Research, College of Medicine, King Saud University, Saudi Arabia

## Abstract

**Objectives:**

This papers aims to investigate the association between different levels of postpartum weight retention (PPWR) and cardiometabolic risk among the Saudi women 12 months postpartum.

**Methods:**

This study is a follow-up of subgroup of cohort from Riyadh mother and baby multicenter cohort study. Clinical data were collected from participants 12 months after delivery and included current Body Mass Index (BMI), waist circumference, hip circumference, and blood pressure. In addition the following blood tests done were fasting blood glucose (FBG), glycosylated haemoglobin (HbA1c) levels, and lipids profile to assess the participants' cardiometabolic risks. The participants were categorized into three groups based on the level of PPWR: weight retention < 3kg; weight retention 3 to < 7kg; and weight retention ≥ 7kg. Subsequently, the prevalence of cardiometabolic risk factors was compared in the three groups to assess the association between different levels of PPWR and cardiometabolic risk factors. Logistic regression was used to test the effect of PPWR in the development of metabolic syndrome and Adjusted Odds Ratio (AOR) was calculated.

**Results:**

A total of 115 women participated in this study. Around 35% of the study population retained ≥ 7 kg of weight. The prevalence of cardiometabolic risk factors, including metabolic syndrome (MetS), increases with the increase of PPWR (p<0.01). The prevalence of MetS is 13% with highest frequency in the group with the highest weight retention. The determinants developing MetS were prepregnancy weight; AOR (95% CI); 1.08 (1.02-1.14), P< 0.01, current BMI, AOR (95% CI); 1.30 (1.12-1.51), P< 0.01, and FBG during pregnancy, AOR (95% CI); and 4.82 (1.72-13.48), P < 0.01.

**Conclusion:**

Increased weight retention after delivery augments the rate of occurrence of cardiometabolic risk factors. Determinants of the development of MetS in postpartum Saudi women are increased prepregnancy weight, current BMI, and FBG during pregnancy.

## 1. Introduction

Excess maternal gestational weight gain (GWG) outside the recommendations of Institute of Medicine (IOM) is associated with increased postpartum weight retention (PPWR) and increased Body Mass Index (BMI). Normal weight and overweight women with GWG above the recommended levels by IOM have nearly threefold increased risk of retaining more than five kilograms compared to those with GWG within the recommendations [1]. Many studies linked PPWR with increased postpartum obesity and central adiposity which in turn is associated with the development of hypertension, dysglycemia, metabolic syndrome (MetS), and other cardiometabolic risk factors [2]. Recent reports from Europe showed that women are at greater risk of fatal and nonfatal cardiovascular disease (CVD) with fivefold increase in the prevalence of metabolic syndrome among women compared to twofold increase in men with increase in age from below 40 years to 60 years and above [3]

Saudi Arabia, one of the high income countries in the Middle East, has witnessed rapid urbanization over the last few decades with simultaneous increase in the burden of CVD [4]. According to the World Health Organization (WHO) country profile of Saudi Arabia, 73% of the accounted deaths were due to noncommunicable diseases, of which 37% were attributed to CVD [5]. The same report showed that obesity and sedentary lifestyle were among the leading risk factors for CVD in the country [5]. In addition, conditions such as hypertension and diabetes mellitus and behaviors such as smoking were recognized as risk factors for CVD in Saudi Arabia [6–10]

Consistent reports from RAHMA study in Saudi Arabia described high rates of obesity, gestational diabetes (GDM), and pregestational diabetes among pregnant women reflecting on growing concern due to the association of these conditions with CVD [11–13]. Recent meta-analysis of the prevalence of cardiometabolic risk factors among Saudi women showed a high prevalence of obesity, diabetes, and physical inactivity [14]

Prevention of CVD is feasible with health education and lifestyle modifications especially when postpartum women are specifically targeted as high risk group for CVD [15, 16]. Hence, the investigation of GWG and PPWR and their impact on maternal cardiometabolic profile among Saudi women will provide important information for primary prevention of CVD in a large sector of the community.

The objective of this study is to investigate the association between different levels of PPWR and cardiometabolic profile among the Saudi women 12 months postpartum.

## 2. Methods

### 2.1. Study Design and Setting

This study is a follow-up of subgroup of cohort from Riyadh mother and baby multicenter cohort study (RAHMA) [17]. RAHMA is a hospital-based prospective cohort study conducted in Riyadh, Saudi Arabia. The study is the first large multicenter, longitudinal cohort study which investigates pregnancy outcomes in Saudi Arabia. The main objectives of the study were to examine the influence of noncommunicable diseases such as diabetes, hypertension, and obesity, on the mother and the baby. It recruited pregnant women and their newborn from three hospitals representing ministry of health, military, and university hospitals [11]. The detailed methodology of the study has been previously reported [11]. This follow-up study was conducted in a subcohort of postpartum women from King Khalid University Hospital (KKUH). During the study period January 2016 to June 2016 all women who participated in RAHMA study from KKUH and met the inclusion criteria were contacted one year after each woman's delivery date to participate in the study. The inclusion criteria for this subcohort were the following.

(1) Gestational age of 37-41 weeks at the time of delivery, calculated from the last menstrual period and/or early ultrasound scan.

(2) Singleton pregnancy.

We excluded women with unknown glycemic status, those with preexisting diabetes or hypertension, diagnosed before or during pregnancy, women who were pregnant at the time of this study, and those who declined to participate in the study.

Women who agreed to participate were invited to attend RAHMA outpatient clinic (research clinic) fasting for at least eight hours before their appointment. The objectives of the study were explained again face-to-face to each participant who subsequently signed an informed consent form. Previously collected data from RAHMA registry for each participant were linked to this study data, including demographic profile, obstetric history such as parity, and prepregnancy weight. Clinical data measured in each participant included current weight and height to calculate the Body Mass Index (BMI) in addition to waist circumference (WC), hip circumference (HC), and systolic and diastolic blood pressure. Blood tests done for each participant were fasting plasma glucose (FPG), HbA1c levels, and lipids profile. The blood glucose level was measured by the glucose oxidase/peroxidase method, and the blood lipids levels were assessed using the esterase oxidase/peroxidase method for serum cholesterol and the glycerokinase oxidase/peroxidase method for HDL, LDL, and triglycerides.

To facilitate comparison, these data were linked to previous data from the RAHMA registry recorded during the initial antenatal visits.

Postpartum weight retention was calculated by subtracting the prepregnancy weight from the postpartum weight at the end of one year from delivery. The study population was then categorized into three groups on the basis of weight retention: women with weight retention <3kg; women with weight retention 3 to less than 7kg; and those with weight retention ≥ 7kg.

### 2.2. Sample Size

The sample size was calculated based on an expected prevalence of MetS among different weight retention groups of 6-25%, with a level of significance of 95% (alpha =0.05), and power of 80% (beta =0.02), and the minimal sample size required to reject the null hypothesis as calculated by G-Power software is 112 [18].

## 3. Definitions

### 3.1. Metabolic Syndrome (MetS)

National Cholesterol Education Program-Adult Treatment Panel III (NCEP-ATP III) criteria were adopted for the diagnosis of MetS which is defined as the presence of any three or more of the following risk factors [19]:

1. fasting blood glucose greater than 5.6 mmol/L (110 mg/dl) or drug treatment for elevated blood glucose.
2. HDL cholesterol < 1.0 mmol/L (40 mg/dl) in men, < 1.3 mmol/L (50 mg/dl) in women or drug treatment for low levels of High-Density Lipoprotein-C (HDL-C)
3. Blood triglycerides (TG) > 1.7 mmol/L (150 mg/dl) or drug treatment for elevated triglycerides
4. Waist circumference > 102 cm (men) or > 88 cm (women)
5. Blood pressure > 130/85 mmHg or drug treatment for hypertension

### 3.2. Dyslipidemia

Adult treatment panel III criteria (ATP III) defines dyslipidemia using the following; raised level of total cholesterol (TC> 200 mg/dL) or Low-Density Lipoprotein –C (LDL-C > 130 mg/dL) or Triglycerides (TG >150 mg/dL) and lower levels of High-Density Lipoprotein-C (HDL-C <50 mg/dL) [19].

### 3.3. Body Mass Index (BMI)

BMI was recorded twice; a prepregnancy BMI was calculated at the first antenatal clinic visit and subsequently the second reading at 1 year postpartum using the formula; maternal weight in kilograms by height in meter^2^ (kg/m^2^)^.^ The study population was then categorized according to WHO definition as follows: underweight (<18.5 kg/m^2^), normal weight (18.5–24.9 kg/m^2^), overweight (25.0–29.9 kg/m^2^), and obese (≥ 30 kg/m^2^) [17].

### 3.4. Gestational Diabetes (GDM)

GDM was diagnosed based on one abnormal value of the 75g oral glucose tolerance test (OGTT) done between 24 and 34 gestation weeks using the following WHO cut-off values [20]:

  FPG= 5.1–6.9 mmol/l (92–125 mg/dl).
  1-hour plasma glucose ≥ 10.0 mmol/l (180 mg/dl).
  2-hour plasma glucose 8.5–11.0 mmol/l (153–199 mg/dl).

### 3.5. Diabetes

FPG or glycosylated hemoglobin (HbA1c) levels according to American Diabetes Association (ADA) criteria were used to diagnose diabetes and prediabetes [21]:

*Fasting Plasma Glucose Values*

  Normal < 5.6 mmol/l (100 mg/dl).
  Prediabetes 5.6-6.9mmol/l (100 mg/dl to 125 mg/dl).
  Diabetes ≥ 7.0 mmol/l (126 mg/dl).

*HbA1c Values*

  Normal < than 5.7% (39 mmol/mol)
  Prediabetes 5.7% to 6.4% (39 to 46 mmol/mol)
  Diabetes≥ 6.5% (48 mmol/mol)

### 3.6. Statistical Analysis

Statistical analysis was performed using SPSS version 21.0 (SPSS Inc., Chicago, IL, USA) for Windows®. Descriptive statistics in terms of average ± Standard deviation for quantitative variables and frequency and percentage for qualitative variables were used. One-way Analysis of Variance (ANOVA) was used to compare quantitative variables among the three groups of weight retention after testing the normality distribution and Kruskal Wallis test was used if data were found skewed. Chi-Square test was used to evaluate the association between categorical variables and Fisher's Exact test was used when indicated; P value <0.05 was considered statistically significant.

Logistic regression model was developed to test the effect of weight retention in developing MetS by calculating the Adjusted Odds Ratio (AOR). The model considered the development of MetS as a binary outcome and it was adjusted for clinically significant confounders including maternal age, prepregnancy weight, current BMI, and FBG during pregnancy.

### 3.7. Ethical Approval

Ethical approval was obtained from the Institution Review Board of King Saud University with approval letter number 15/0445/IRB. A written consent was signed by every participant prior to the start of study after explaining the procedures involved.

## 4. Results

A total of 115 women met the inclusion criteria and consented to participate in this study. Thirty-four women had weight retention <3kg and 40 women had weight retention 3 to < 7kg while 41 had weight retention ≥ 7kg one year after delivery. The demographic and maternal characteristics are shown in Table 1. The mean age was similar across the three groups. Although not statically significant, university education and employment were more frequent in the groups with lesser weight retention. Women with the highest weight retention had significantly higher prepregnancy mean body weight compared to women who retained less weight (p<0.01). In addition, the fasting mean blood glucose levels on OGTT during pregnancy showed an incremental increasing value across the three groups, with highest mean values observed in women with** ≥** 7kg weight retention (p=0.03) (Table 1). Other characteristics were not significantly different between the groups (Table 1).

Table 2 shows the comparison of the frequency of cardiometabolic risk factors among the three postpartum weight retention groups. An increasing trend in prevalence of metabolic risk factors is observed with increasing weight retention. The prevalence of MetS in the study group was 13% with highest frequency in the group with the highest weight retention (Table 2).

In addition, the group with highest weight retention had highest prevalence of hyperglycemia (p=0.02) and central obesity (p=0.02) compared to the other two groups. Although not significant, the prevalence of hypertension also increased with increasing postpartum weight retention.

**Figure 1:**
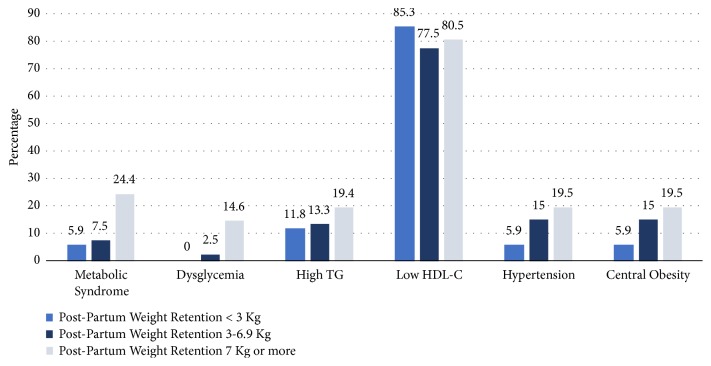
*Prevalence of cardiometabolic risk factors according to different groups of weight retention one-year postpartum*. High TG=triglyceride > 1.7 mmol/L, low HDL-C= high density lipoprotein cholesterol< 1.0 mmol/L in men, < 1.3 mmol/L in women, dysglycemia=fasting blood glucose greater than 5.6 mmol/L, and metabolic syndrome according to Adult Treatment Panel III.

The relationship between the prevalence of cardiometabolic risk factors and the level of PPWR is shown in Figure 1, which shows an incremental increase in prevalence of cardiometabolic risk factors with the increase of PPWR in a step-ladder fashion, which indicates that the increase in PPWR is associated with the increased prevalence of cardiometabolic risk factors. Low levels of HDL-Cholesterol were prevalent in all the three groups.

The result of the regression analysis showed that the main determinants for the development of MetS in Saudi postpartum women were prepregnancy weight, current BMI, and the mean fasting blood glucose during pregnancy (Table 3).

## 5. Discussion

The results of this study showed that 35% of the study population had retained seven or more kilograms of weight and that the prevalence of cardiometabolic risk factors, including MetS, increases with the increase of PPWR. In addition, the study showed that the prevalence of MetS among all the study participants is 13% and the main determinants of the development of MetS in postpartum Saudi women are prepregnancy weight, current weight, and fasting plasma glucose level during pregnancy.

Previous reports showed that PPWR is influenced by prepregnancy weight and GWG [22, 23]. Women who are obese or overweight prior to pregnancy have nearly twice the risk of excessive GWG compared to those with normal prepregnancy weight [24]. Furthermore, excessive GWG increases the risk of excessive PPWR and hence increases the cardiometabolic risk factors [25–27]. Moreover, studies showed that postpartum weight loss and maintenance of the loss is associated with noticeable improvement in cardiometabolic profile [28, 29]. These findings are consistent with the findings about the detrimental effects of weight gain in young adults, on the cardiovascular risk irrespective of gender or race [30].

Recently published studies showed high prevalence of 35-44% of obesity among Saudi women [6, 11]. In addition, maternal obesity is found to be a strong predictor of adverse pregnancy outcomes in Saudi women [31] and is associated with postpartum dysglycemia [13, 32].

Similar to our findings, all the range of glucose intolerance during pregnancy, including GDM, was found to be associated with increased risk of developing cardiometabolic risk factors in postpartum women in a gradient fashion [24, 33]. Published reports from Saudi Arabia showed that nearly 30% of pregnant women have pregestational or gestational diabetes mellitus, which puts considerable proportion of women in reproductive age at increased risk of developing CVD [12].

The prevalence of 13% for MetS for the total study population is consistent with the lower level reported for Saudi population in general and specifically for Saudi women [14, 34]. However, this prevalence is higher than that reported for a similar age group of women in Europe [35]. Globally the prevalence of MetS and different clusters of cardiometabolic risk factors is influenced by race, gender, and age [3, 35].

It is noticeable that high proportion of each of the three groups of participants had low HDL-cholesterol level (Figure 1). This observation could be considered as part of the physiological alternation in the lipid metabolism during pregnancy which has been reported in earlier studies [36–38] and which serves to fulfill the increase in mother and fetus energy needs. During pregnancy women show an incremental increase in triglyceride and total cholesterol serum levels with the progression of pregnancy, compared to those who are not pregnant, with the highest levels during the third trimester [37]. During the postpartum period there is marked drop of all lipid levels; however, it was observed that, one year after delivery, HDL-cholesterol levels dropped even below prepregnancy concentrations [38], which concurs with our observation in the current study.

Recently the burden of noncommunicable diseases has increased in the Arab World, including Saudi Arabia, with noticeable shift from infectious diseases to chronic diseases, such as cardiovascular diseases, as a cause of morbidity, mortality, and premature death [39, 40]. This shift in the burden of disease calls for scaling-up the detection of high risk group, including postpartum women, as target for evidence-based preventive interventions.

Lifestyle modification starting during the preconception or antenatal period has been associated with moderate reduction in PPWR [41, 42]. Based on our findings that BMI is a risk factor for both MetS and CVD, preconception and antenatal lifestyle interventions may be a valid intervention to reduce the cardiometabolic risks in women of reproductive age group in Saudi Arabia. However such intervention needs robust antenatal program not only for the detection of women at high risk for CVD but also to provide the motivation for women to change their dietary habits and practice regular exercise [43]. Lifestyle modification in the postpartum period and in between pregnancies is another effective strategy to reduce weight GWG, obesity during pregnancy, and PPWR [44]

Another proven preventive measure for CVD risk is longer period of breast feeding which was proven to reduce the risk of developing MetS especially in women who breastfeed their children nine months or more [45, 46]. However, we did not find significant difference between the three groups in the prevalence of initiation or maintenance of breast feeding.

## 6. Strength and Limitations

This follow-up study of a subgroup of RAHMA study is the first in Saudi Arabia to explore the incidence of cardiometabolic risk factors in postpartum women. The evidence provided by this study can be used in conjunction with other epidemiological data and studies for planning of preventive strategy for CVD for pregnant and postpartum women who are utilizing the health services, hence reducing the cost of screening. The study is a successful attempt of a longitudinal cohort study. Such studies are difficult to conduct due to the many cultural and logistic barriers in the Middle East.

We are aware of the limitation of this study including the small number of participants and the inclusion of participants from only one center out of the three centers of the main cohort; both conditions may limit the generalization of the results to the community of postpartum women in Riyadh. In addition, the study may have overestimated the prevalence of the cardiometabolic risks based of the possibility of selection bias of women who agreed to participate in the study rather than all the women who participated in RAHMA cohort.

## 7. Conclusion

More than one-third of postpartum women have retained seven or more kilograms of gestational weight. That prevalence of cardiometabolic risk factors, including MetS, increases with the increase of PPWR. The prevalence of MetS is 13% among all the participants of the study and the main determinants of the development of MetS in postpartum Saudi women are prepregnancy weight, current weight, and fasting plasma glucose level during pregnancy.

## Figures and Tables

**Table 1:** Comparison of the demographic and obstetric characteristics of the study population according to postpartum weight retention category.

Characteristic	Weight retention <3kg N=34	Weight retention 3 to less than 7kg N=40	Weight retention ≥ 7kg N=41	p-value
Age	31.2±5.7	31.9±5.3	32.2±6.4	0.75

*Education*				

Schools	12 (35.3)	13 (32.5)	17 (41.5)	0.69
University or higher	22 (64.7)	27 (67.5)	24 (58.5)	

*Occupation*				

Housewife	27 (79.4)	33 (82.5)	36 (87.8)	0.61
Employed	7 (20.6)	7 (17.5)	5 (12.2)	

*Monthly income*				

<5,000 SR	2 (6.1)	1(2.6)	1(2.5)	0.61
5,000-<10,000 SR	18 (54.5)	19 (50.0)	26 (65.0)	
10,000 or more SR	13 (39.4)	18 (47.4)	13 (32.5)	

*Family history of diabetes*	19 (55.9)	17(42.5)	23 (56.1)	0.39

*Index Pregnancy characteristics*				

Pre-pregnancy weight(kg)	65.4±12.8	65.0±10.3	72.6±11.9	*<0.01* ^¶^

Parity	2.6±2.0	3.2±1.6	2.9±1.6	0.37

OGTT results (mmol/l)				

Fasting plasma glucose	4.4±0.5	4.5±0.5	4.7±0.7	*0.03* ^**§**^

One-hour plasma glucose	7.7±1.5	7.1±1.8	7.3±0.7	0.38

Two-hour plasma glucose	6.1±1.7	5.9±1.6	5.9±1.9	0.92

GDM	3 (8.8)	5 (15.0)	8 (19.5)	0.43

Pregnancy associated HTN	1 (2.9)	2 (5.0)	2 (4.8)	0.31

Caesarean section delivery	6 (17.6)	9 (22.5)	12 (29.3)	0.20

Birth weight	2.8±0.4	2.8±0.3	2.9±0.3	0.63

*Postpartum*				

Breast feeding				

Initiation	29 (85.3)	39 (97.5)	40 (97.6)	0.06

At 4 months	20 (58.8)	25 (62.5)	27 (65.9)	0.82

At 6 months	16 (47.1)	12 (30.0)	13 (31.7)	0.25

At 12 months	7 (20.6)	2 (5.0)	7 (17.1)	0.11

Contraception				

Hormonal	16 (47.1)	22 (55.0)	21 (51.2)	0.778
Non-hormonal	4 (11.8)	7 (17.5)	7 (17.1)	

GDM = Gestational diabetes, OGTT = Oral glucose tolerance test, HTN=Hypertension, ^¶^significant difference between participants in Weight retention ≥ 7kg and the other two groups, ^**§**^significant difference between participants in Weight retention ≥ 7kg and participants with Weight retention <3kg

**Table 2:** Comparison of cardio-metabolic risk factors among women with different postpartum weight retention levels.

Mean values of biochemical and anthropometric parameters (Mean ±SD)	Weight retention <3kg N=34	Weight retention 3 to < 7kg N=40	Weight retention ≥ 7kg N=41	p-value
HbA1c(%)	5.4±0.4	5.3±0.4	5.5±0.4	0.18

LDL-C (mg/dL)	2.7±0.6	2.6±0.7	2.8±0.8	0.49

Total Cholesterol(mg/dL)	4.3±0.7	4.2±0.8	4.40.8±	0.38

Current BMI (kg/m^2^)	25.9±5.1	28.2±4.3	33.6±4.9	*<0.01* ^§^

Hip circumference (cm)	95.5±9.4	95.1±17.6	104.2±18.8	*0.02*

Prevalence of cardio-metabolic risk factors				

Metabolic Syndrome	2 (5.9)	3 (7.5)	10 (24.4)	*0.03*

Hyperglycemia	0 (0.0)	1(2.5)	6 (14.6)	*0.02*

Central Obesity	5 (14.7)	8 (20.0)	17 (41.5)	*0.02*

Abnormal TG	4 (11.8)	6 (13.3)	7 (19.4)	0.62

Abnormal HDL-C	29 (85.3)	31(77.5)	33 (80.5)	0.69

Hypertension	2 (5.9)	6 (15.0)	8 (19.5)	0.23

Data expressed as either mean± SD or n (%), TG=Triglyceride, HDL-C= High density lipoprotein cholesterol,

LDH-C= Low density lipoprotein cholesterol,^** §**^significant difference between participants in Weight retention ≥ 7kg and participants with Weight retention <3kg

**Table 3:** Risk factors for the development of metabolic syndrome.

Risk factor	OR (95% C.I.)	p-value	AOR (95% C.I.)	p-value
Postpartum weight retention				

<3 kg	1		1	

3 to less than 7 kg	1.30 (0.20-8.26)	0.78	1.53 (0.23-10.27)	0.66

≥ 7 kg	5.16 (1.05-25.48)	0.04	3.71 (0.71-19.42)	0.12

Pre-pregnancy weight(kg)	1.09 (1.03-1.14)	<0.01	1.08 (1.02-1.14)	<0.01

Current BMI(kg/m^2^)	1.30 (1.14-1.49)	<0.01	1.30 (1.12-1.51)	<0.01

Fasting Blood glucose during pregnancy(mmol/l)	6.06 (2.21-16.65)	<0.01	4.82 (1.72-13.48)	<0.01

P<0.05 indicates a significant interaction term in the logistic regression model.

Adjusted for category of weight retention, current BMI, Pre-pregnancy weight and FBG during pregnancy.

Metabolic syndrome defined by the National Cholesterol Education Program - Adult Treatment Panel III

## Data Availability

The data used to support the findings of this study are available from the corresponding author upon request.
